# Complete Response After One Cycle of Atezolizumab Plus Bevacizumab in Advanced Hepatocellular Carcinoma: Two High-Risk Contexts, Child-Pugh Class B and Advanced Age

**DOI:** 10.7759/cureus.97689

**Published:** 2025-11-24

**Authors:** Masamichi Kimura, Yumi Otoyama, Koji Nishikawa, Jun Imamura, Kiminori Kimura

**Affiliations:** 1 Hepatology, Tokyo Metropolitan Cancer and Infectious Diseases Center, Komagome Hospital, Tokyo, JPN

**Keywords:** atezolizumab plus bevacizumab, atezolizumab plus bevacizumab therapy, hepatocellular carcinoma (hcc), immune-checkpoint-inhibitor, liver hepatitis hepatocellular carcinoma

## Abstract

Complete responses after one cycle of atezolizumab-bevacizumab have been described, but evidence in two high-risk contexts - patients with Child-Pugh class B liver function and very old adults - is scarce. We report two illustrative cases of advanced hepatocellular carcinoma where radiographic complete response was observed after a single treatment cycle. Case 1 involved a patient with Child-Pugh class B liver function; Case 2 involved an older adult. For both cases, we summarize baseline characteristics, serial imaging, and tumor-marker trajectories that corroborate response after minimal exposure to therapy. Radiologic responses were assessed on multiphasic contrast-enhanced CT according to mRECIST; tumor-marker declines supported but did not determine the classification. These two cases do not establish efficacy or safety in these populations; rather, they are descriptive observations showing that such responses can occasionally occur after one treatment cycle and should be interpreted with appropriate caution. Given limited data and safety considerations in Child-Pugh class B and very old patients, the findings should not be generalized without further validation.

## Introduction

Hepatocellular carcinoma (HCC) often develops in the background of chronic hepatitis or cirrhosis [1]. In advanced cases, systemic therapies centered on molecularly targeted agents and immune checkpoint inhibitors are used. The combination of atezolizumab (Atz), an immune checkpoint inhibitor, and bevacizumab (Bev), an angiogenesis inhibitor, is a preferred first-line systemic option for unresectable/advanced HCC, as established in the phase III IMbrave150 trial [2,3]. Its response patterns are characterized [4], and the combination has been endorsed by major guidelines [5]. However, prospective efficacy and safety data remain limited in older adults and in those with compromised hepatic reserve (e.g., Child-Pugh class B); notably, IMbrave150 only enrolled cases of Child-Pugh class A, and real-world analyses in class B report attenuated outcomes and higher rates of serious adverse events, warranting careful, individualized eligibility assessments [6-10].

Treatment eligibility hinges on hepatic reserve, so the Child-Pugh classification, comprising serum bilirubin, albumin, prothrombin time/international normalised ratio (INR), ascites, and hepatic encephalopathy, stratifies patients into classes A (5-6 points), B (7-9), or C (10-15). Pivotal immunotherapy trials, including IMbrave150, have largely restricted enrollment to class A [2,3]; patients with class B are underrepresented and often excluded, leaving key uncertainties regarding benefit-risk in this group [6-10]. Against this background, we present two illustrative cases where radiographic complete response (CR) was observed after one cycle of Atz-Bev in high-risk contexts: (i) Child-Pugh class B liver function and (ii) advanced age. By highlighting these outcomes, this report aims to inform clinical decision-making for patients who fall outside the conventional trial-eligibility criteria.

## Case presentation

Case 1

A 66-year-old man with a remote history of hepatitis B infection without regular follow-up developed gastric discomfort. At another hospital, contrast-enhanced abdominal CT obtained for these symptoms demonstrated a hepatic mass compatible with hepatocellular carcinoma and imaging features of cirrhosis, prompting referral to our center for further evaluation and treatment. He had chronic hepatitis B-related cirrhosis and had been maintained on entecavir prior to presentation; antiviral therapy was continued during radiotherapy and subsequent Atz-Bev to reduce the risk of HBV reactivation. He had no other significant comorbidities, but reported a 40-year history of smoking approximately 20 cigarettes per day and consuming one 350 mL can of beer daily. His height was 172.9 cm and weight 69.8 kg (body mass index 23.3 kg/m²). He was unemployed during the presentation. There was no family history of cancer or liver disease. On admission, he had mild abdominal distention without tenderness.

**Figure 1:**
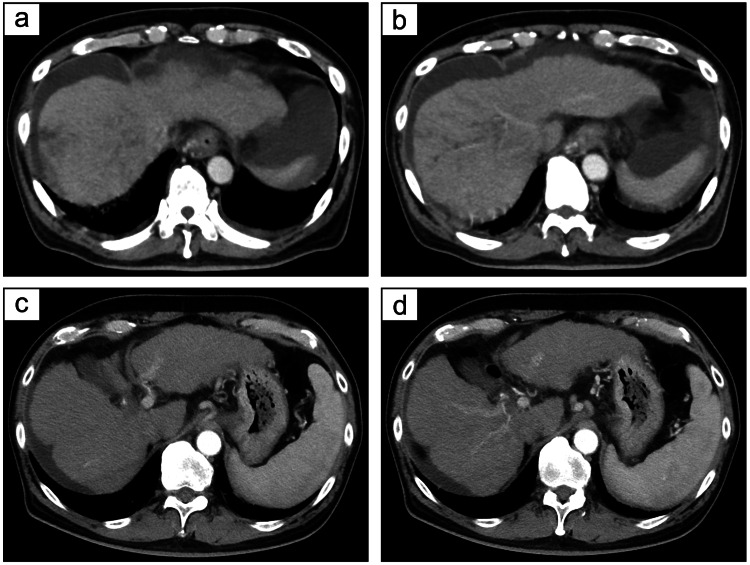
Contrast-enhanced computed tomography scan before and after treatment (a, b) Contrast-enhanced computed tomography (CT) showing a 37-mm mass in segment 8 of the liver with a tumor thrombus in the right portal vein branch. The lack of early contrast enhancement in the dynamic study suggests poorly differentiated hepatocellular carcinoma. (c, d) Follow-up contrast-enhanced CT images showing disappearance of the segment 8 liver mass, indicating a complete response.

CT scans revealed ascites and a 37-mm mass in segment 8 of the liver, along with multiple hypoattenuating lesions suggestive of intrahepatic metastases (Figure 1A, 1B). Tumor thrombus was noted in the right portal vein branch, but no lymph node or distant metastases were observed. Laboratory findings showed a prothrombin time (PT) of 81%, albumin of 2.7 g/dL, and total bilirubin of 0.6 mg/dL (Table 1). His Child-Pugh classification was B (7 points), and the albumin-bilirubin (ALBI) score was −1.63 (Grade 2b). CRP was 1.95 mg/dL (≈19.5 mg/L). In the absence of documented fever or localizing infectious symptoms, this mild-moderate elevation was considered most compatible with tumor-related systemic inflammation in the setting of a large hepatic mass and portal vein tumor thrombus (PVTT), while alternative causes (e.g., cholangitis/biliary obstruction, spontaneous bacterial peritonitis, pneumonia, urinary tract infection, drug-related inflammation) were considered and clinically monitored.

**Table 1:** Laboratory Blood Examination Results on Admission Abbreviations: WBC: white blood cell, RBC: red blood cell, Hb: hemoglobin, Plt: platelet, PT-INR: prothrombin time-international normalized ratio, APTT: activated partial thromboplastin time, BUN: blood urea nitrogen, Cre: creatinine, AST: aspartate aminotransferase, ALT: alanine aminotransferase, γ-GTP: γ-glutamyltransferase, ALP: alkaline phosphatase, T-Bil: total bilirubin, LDH: lactate dehydrogenase, CRP: C-reactive protein, AFP: alpha-fetoprotein, PIVKA-II: protein induced by vitamin K absence-II, HBsAg: hepatitis B surface antigen, HBsAb: Hepatitis B surface antibody, HBcAb: hepatitis B core antibody, HCVAb: hepatitis C virus antibody

Laboratory parameter	Results	Normal values
WBC (/μL)	7,800	3,300–8,600
RBC (/μL)	233 × 10^4^	435–555× 10^4^
Hb (g/dL)	7.6	13.7–16.8
Plt (/μL)	17.5 × 10^4^	15.8–34.8× 10^4^
PT-INR	1.13	0.85–1.15
Albumin (g/dL)	2.7	3.8–5.2
BUN (mg/dL)	20	8–20
Cre (mg/dL)	0.78	0.65–1.07
AST (U/L)	74	13–30
ALT (U/L)	75	10–42
LDH (U/L)	304	124–222
ALP (U/L)	161	38–113
γ-GTP (U/L)	326	13–64
T-Bil (mg/dL)	0.6	0.4–1.5
Na (mmol/L)	141	138–145
K (mmol/L)	3.9	3.6–4.8
CL (mmol/L)	107	101–108
CRP (mg/dL)	1.95	0–0.14
HBsAg	Positive	Negative
HBsAb	Positive	Negative
HBcAb	Positive	Negative
HBV-DNA (Log IU/mL)	2.7	Negative
HCVAb	Negative	Negative
AFP (ng/mL)	13.7	< 10.0
PIVKA-II (mAU/mL)	34,330	< 40

Given the Child-Pugh class B (7 points) with a serum albumin of 2.7 g/dL, borderlining the 2.8 g/dL threshold within the Child-Pugh score and an ALBI score of −1.63 (grade 2b) in the presence of PVTT, our multidisciplinary team discussed management options including best supportive care, immediate systemic therapy, and a radiation-first approach to the PVTT. Anticipating that the local control of the thrombus could stabilize or potentially improve hepatic reserve, risks (hepatic decompensation, bevacizumab-related bleeding, immune-related adverse events) and alternatives were reviewed with the patient. By shared decision-making, the patient preferred an active anti-tumor strategy; therefore, radiotherapy to the PVTT was performed before initiating systemic therapy. One cycle of Atz plus Bev was administered with close monitoring.

Elevated tumor markers included PIVKA-II at 34,330 mAU/mL and alpha-fetoprotein (AFP) at 13.7 ng/mL.

**Figure 2:**
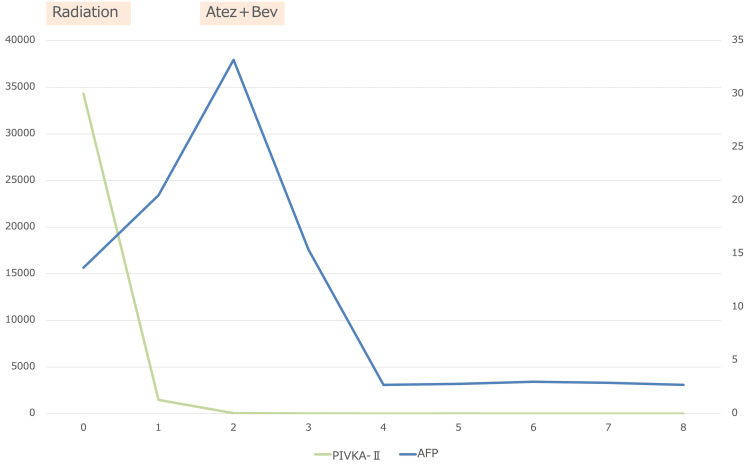
Temporal changes in tumor markers (AFP and PIVKA-II) in Case 1 The graph shows serial alpha-fetoprotein (AFP; left y-axis, ng/mL) and PIVKA-II (right y-axis, ×10³ mAU/mL) over time (x-axis, months from baseline). PIVKA-II rapidly declined from ~34×10³ mAU/mL to within the normal range by Month 2 and remained suppressed thereafter, while AFP fell from a peak of ~38,000 ng/mL to low levels by Month 4 with subsequent stabilization. Radiotherapy to portal vein tumor thrombus preceded one cycle of atezolizumab plus bevacizumab, as described in the case narrative.

Therapeutically, the patient first underwent radiation therapy (30 Gy/10 fractions) targeting the portal vein tumor thrombus, followed by the initial administration of atezolizumab-bevacizumab. However, 3 weeks later, during an outpatient visit for the planned second infusion, worsening ascites and bilateral lower-leg edema were noted, leading to the discontinuation of therapy. A CT scan performed five months after the first infusion revealed complete tumor disappearance, confirming a CR (Figure 1C, 1D). His tumor markers also normalized, and the CR status has since been maintained (Figure 2).

Case 2

A 79-year-old woman under follow-up at another hospital for osteoporosis was referred to our institution after routine follow-up blood tests revealed abnormal liver biochemistry, prompting an abdominal ultrasound that detected a hepatic tumor. Her medical history included osteoporosis; otherwise, it was unremarkable, with no history of smoking or alcohol consumption. Her height was 148.2 cm and weight 56.9 kg (body mass index 25.9 kg/m²). Her occupational history was non-contributory, and there was no family history of liver disease.

**Figure 3:**
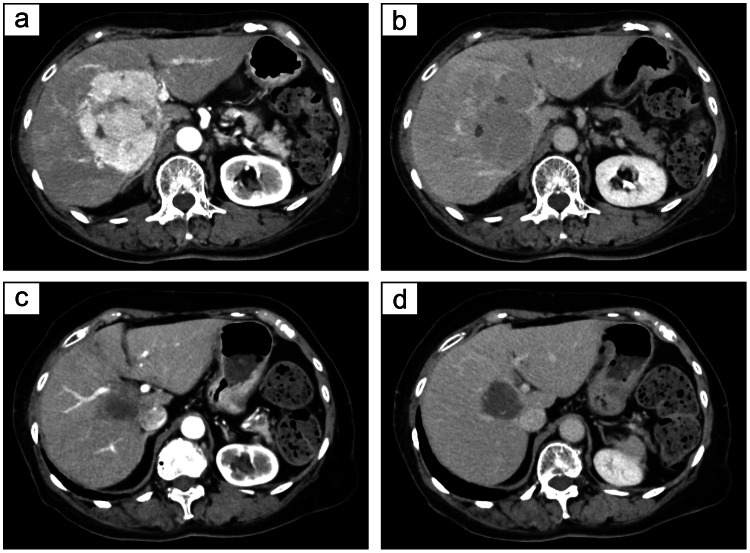
Contrast-enhanced CT scan before and after treatment (a, b) Contrast-enhanced CT scans showing an 8 cm mass in segment 6 of the liver, with a portion of the lesion exhibiting viable tumor tissue. (c, d) Follow-up contrast-enhanced CT showing a reduction in the segment 6 liver mass, with the previously noted viable portion no longer visible.

A CT scan identified an 8-cm tumor in segment 6 of the liver (Figure 3). Laboratory results showed PT-INR 0.94, albumin 4.1 g/dL, and total bilirubin 1.2 mg/dL (Table 2), corresponding to Child-Pugh class A (5 points) and an ALBI score of −2.64 (Grade 1), indicating preserved hepatic function. Elevated tumor-marker levels were noted, with AFP at 45.9 ng/mL and PIVKA-II at 121 mAU/mL. CRP was 1.95 mg/dL (≈19.5 mg/L). In the absence of documented fever or localizing infectious symptoms, this mild-moderate elevation was considered most compatible with tumor-related systemic inflammation in the setting of a large hepatic mass, while alternative causes (e.g., cholangitis/biliary obstruction, spontaneous bacterial peritonitis, pneumonia, urinary tract infection, drug-related inflammation) were considered and clinically monitored.

**Table 2:** Laboratory Blood Examination Results on Admission Abbreviations: WBC: white blood cell, RBC: red blood cell, Hb: hemoglobin, Plt: platelet, PT-INR: prothrombin time-international normalized ratio, APTT: activated partial thromboplastin time, BUN: blood urea nitrogen, Cre: creatinine, AST: aspartate aminotransferase, ALT: alanine aminotransferase, γ-GTP: γ-glutamyltransferase, ALP: alkaline phosphatase, T-Bil: total bilirubin, LDH: lactate dehydrogenase, CRP: C-reactive protein, AFP: alpha-fetoprotein, PIVKA-II: protein induced by vitamin K absence-II, HBsAg: hepatitis B surface antigen, HBsAb: Hepatitis B surface antibody, HBcAb: hepatitis B core antibody, HCVAb: hepatitis C virus antibody

Laboratory parameter	Results	Normal values
WBC (/μL)	4,900	3,300–8,600
RBC (/μL)	425 × 10^4^	435–555× 10^4^
Hb (g/dL)	12.3	13.7–16.8
Plt (/μL)	22.6 × 10^4^	15.8–34.8× 10^4^
PT-INR	0.94	0.85–1.15
Albumin (g/dL)	4.1	3.8–5.2
BUN (mg/dL)	17	8–20
Cre (mg/dL)	0.79	0.65–1.07
AST (U/L)	126	13–30
ALT (U/L)	104	10–42
LDH (U/L)	222	124–222
ALP (U/L)	348	38–113
γ-GTP (U/L)	256	13–64
T-Bil (mg/dL)	0.6	0.4–1.5
Na (mmol/L)	142	138–145
K (mmol/L)	4.6	3.6–4.8
CL (mmol/L)	105	101–108
CRP (mg/dL)	0.34	0–0.14
HBsAg	Negative	Negative
HBsAb	Positive	Negative
HBcAb	Positive	Negative
HBV-DNA (Log IU/mL)	Negative	Negative
HCVAb	Negative	Negative
AFP (ng/mL)	45.9	< 10.0
PIVKA-II (mAU/mL)	121	< 40

**Figure 4:**
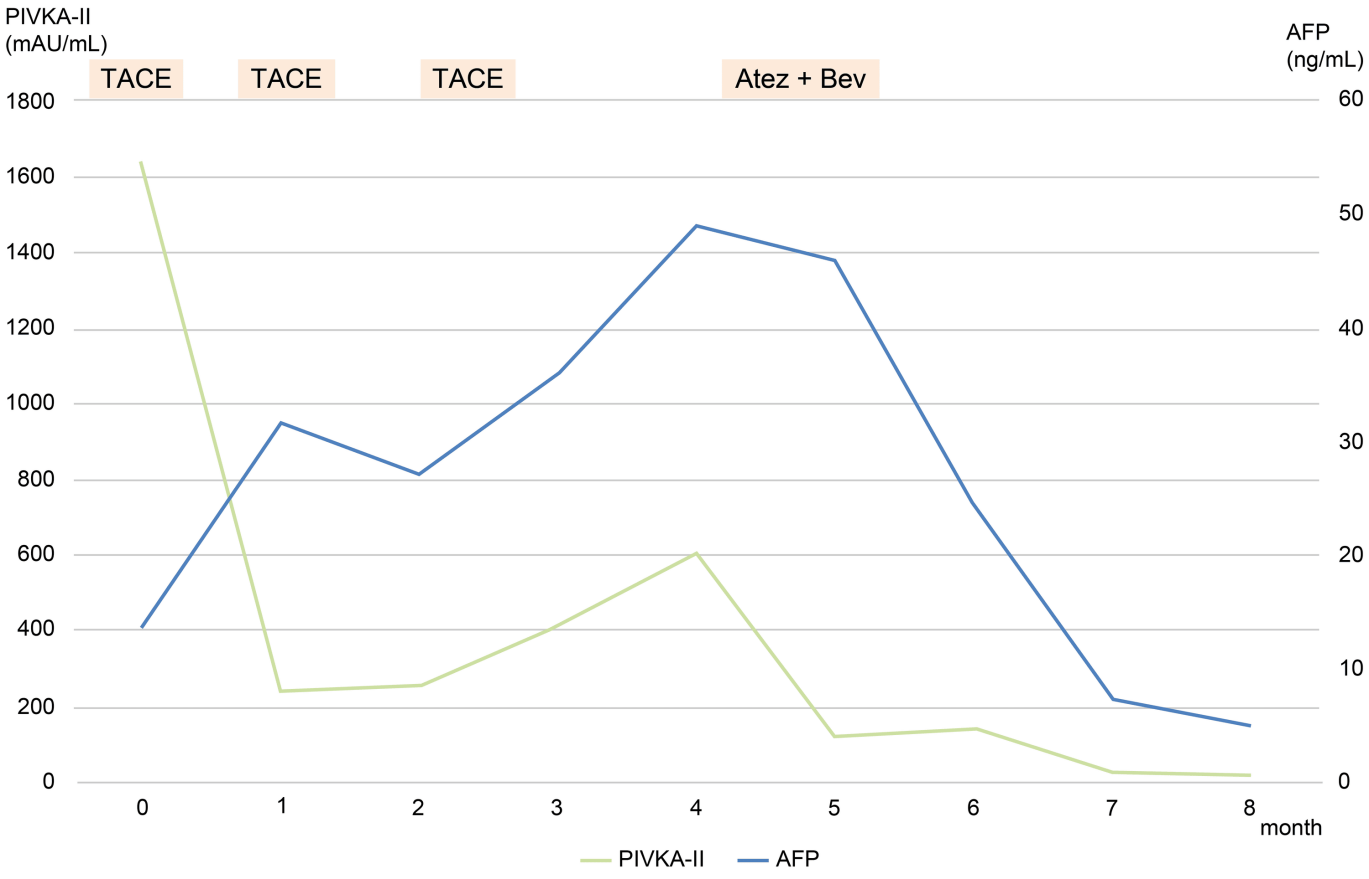
Temporal changes in tumor markers (AFP and PIVKA-II) in Case 2. Serial alpha-fetoprotein (AFP) (ng/mL) and PIVKA-II (mAU/mL) from baseline through four months, demonstrating decline to within institutional reference ranges by one month and sustained suppression at complete response (CR). TACE: transcatheter arterial chemoembolization

A diagnosis of HCC was established non-invasively based on multiphasic contrast-enhanced CT demonstrating arterial-phase hyperenhancement with portal/delayed-phase washout, together with elevated tumor markers (AFP 45.9 ng/mL; PIVKA-II 121 mAU/mL) (Figure 3A, 3B). Given a solitary 8-cm lesion with preserved hepatic reserve (Child-Pugh A5; ALBI grade 1) and no evident macrovascular invasion on baseline imaging, our multidisciplinary team selected initial locoregional therapy with transcatheter arterial chemoembolization (TACE). After three TACE sessions, a viable residual tumor persisted, consistent with TACE refractoriness; therefore, we escalated to systemic therapy with Atz-Bev, reflecting a guideline-endorsed first-line option in eligible patients and the patient’s preference following locoregional failure, with documented shared decision-making and a plan for close monitoring. However, after the first cycle, the patient experienced fever and decreased appetite. Per Common Terminology Criteria for Adverse Events (CTCAE) v5.0, these were classified as pyrexia Grade 1 and decreased appetite Grade 2, and were managed conservatively (antipyretics, oral hydration, nutritional support) with rapid improvement and no infectious focus identified. In shared decision-making, the patient preferred not to re-challenge, and therapy was therefore discontinued before cycle 2. At one month, follow-up CT at matched anatomic levels showed interval tumor shrinkage with decreased arterial-phase hyperenhancement, and AFP and PIVKA-II had fallen to within the institutional reference ranges (Figure 4). At four months, intratumoral arterial enhancement disappeared in all target lesions with no new lesions, consistent with a CR according to mRECIST (Figure 3C, 3D).

## Discussion

The phase III IMbrave150 trial established Atz-Bev as a preferred first-line option for unresectable/advanced HCC and reported CR rates of ~5.5% according to RECIST 1.1 and ~10.2% according to mRECIST, with median time to CR of ~7.0 and ~5.5 months, respectively [2-4]. In contrast, both of our patients achieved radiographic CR after a single treatment cycle: Case 1 at around five months despite PVTT and Child-Pugh class B liver function, and Case 2, an older patient. This illustrates that deep responses can occasionally occur, highlighting heterogeneity.

Because pivotal trials predominantly enrolled Child-Pugh A, real-world cohorts suggest attenuated outcomes and higher rates of grade 3-4 adverse events in Child-Pugh B, creating a clinical dilemma that necessitates individualized risk-benefit assessment, variceal evaluation/management, cautious initiation, and close monitoring [6-10]. HCC is common in older adults, and epidemiologic updates note an increasing burden in this group [11]; although observational analyses often show broadly comparable efficacy and tolerability to younger patients, individual tolerability, as in Case 2, where fever/anorexia prompted early discontinuation, may still limit therapy, underscoring the need for shared decision-making and early supportive care [12-15].

In both cases, radiologic responses were determined on multiphasic contrast-enhanced CT using mRECIST, which requires the disappearance of intratumoral arterial enhancement in all target lesions with no new lesions. Decline in tumor markers supported the classification [16]. Case-specific factors may also have contributed: in Case 1, radiotherapy for PVTT before systemic therapy could have promoted antigen release and immune activation; in Case 2, comparatively preserved hepatic reserve and lack of macrovascular invasion may have facilitated drug delivery and immune infiltration. There was heterogeneity within Child-Pugh B: Case 1 (B7) was driven primarily by low albumin (2.7 g/dL) rather than refractory ascites/encephalopathy, bearing different prognostic/tolerability implications. At our institution, patients with advanced HCC classified Child-Pugh C are generally not candidates for Atz-Bev outside clinical trials; management focuses on symptom control, portal-hypertension care, transplant evaluation where appropriate, and multidisciplinary review. These observations are descriptive and not generalizable, but may inform individualized decisions in carefully selected patients, including those with Child-Pugh B liver function or advanced age. Further prospective data should clarify efficacy and safety in these underrepresented populations.

## Conclusions

These two cases are descriptive and do not justify generalization. They illustrate that radiographic CR after one cycle of Atz-Bev can occasionally occur in carefully selected patients, including those with Child-Pugh class B liver function or advanced age. Treatment decisions should remain individualized with careful risk-benefit assessment, and further prospective data are needed to clarify efficacy and safety in these populations.
